# Supplementary material for: Alpha thalassemia/mental retardation X-linked (ATRX) protein expression in human pituitary neuroendocrine tumours and its reported correlation to prognosis and clinical outcomes: A systematic review
Source: PLoS One. 2025 May 29;20(5):e0313380. doi: 10.1371/journal.pone.0313380 (PMC12121788; doi:10.1371/journal.pone.0313380)
Supplement: File S2 — (PDF) [file pone.0313380.s002.pdf]

Supplementary File 2: List of Review Studies and Statistical Methods

1. Table of Screened Studies

Below is a table of all screened studies in this review. Information regarding authors, year of publication, journal, inclusion/exclusion, and reason for exclusion have been listed in the table.

Table S1: List of Screened Studies

	Authors	Year	Journal	DOI	Included/Excluded	Reason for Exclusion
1	Rindi (Asa)	2022	Endocr Pathol	10.1007/s12022-022-09708-2	Excluded	Wrong study design
2	Sumislawski (Saeger)	2022	Endocrine	10.1007/s12020-021-02954-0	Excluded	Wrong patient population
3	Casar-Borota (Burman)	2021	J Clin Endocrinol Metab	10.1210/clinem/dgaa749	Included	-
4	Burman (Dekkers)	2023	J Clin Endocrinol Metab	10.1210/clinem/dgad098	Excluded	Wrong study design
5	Heaphy (Rodriguez)	2020	Mod Pathol	10.1038/s41379-020-0523-2	Included	-
6	Casar-Borota (Trouillas)	2017	Am J Surg Pathol	10.1097/PAS.0000000000000908	Included	-
7	Chen (Dahiya)	2019	J Neuropathol Exp Neurol	10.1093/jnen/nlz040	Included	-
8	Louis	2018	Brain Tumor Pathol	10.1007/s10014-018-0315-2	Excluded	Wrong study design
9	Agaimy (Klöppel)	2023	Virchows Arch	10.1007/s00428-022-03484-4	Excluded	Wrong patient population
10	Rowland (Bérubé)	2018	Aging (Albany NY)	10.18632/aging.101462	Excluded	Wrong patient population
11	Lu (Chen)	2021	Front Endocrinol (Lausanne)	10.3389/fendo.2021.752361	Included	-
12	Theodoropoulou (Reincke)	2022	Pituitary	10.1007/s11102-022-01253-9	Excluded	Wrong study design
13	Alzoubi (Buttarelli)	2022	Endocr Pathol	10.1007/s12022-021-09702-0	Included	-
14	Guo (Wang)	2018	Front Oncol	10.3389/fonc.2018.00510	Included	-
15	Stojanoski (Casar-Borota)	2021	Front Oncol	10.3389/fonc.2021.739255	Excluded	Wrong patient population
16	Simbolo (Rindi)	2021	Cancers (Basel)	10.3390/cancers13092054	Excluded	Wrong patient population
17	Huang (Lou)	2016	Transl Gastroenterol Hepatol	10.21037/tgh.2016.09.03	Excluded	Wrong patient population

18	Nejo (Mukasa)	2018	J Neurosurg	10.3171/2018.6.JNS18729	Excluded	Wrong patient population
19	Sun (Yang)	2024	Front Oncol	10.3389/fonc.2024.1392610	Excluded	Wrong patient population
20	Wang (Kim)	2023	Familial Cancer	10.1007/s10689-023-00343-2	Excluded	Wrong patient population
21	Hernández Ramírez (Korbonits)	2024	Experimental and Clinical Endocrinology and Diabetes	10.1055/a-2337-2265	Excluded	Wrong study design
22	Lim (Lee)	2019	Endocrine	10.1007/s12020-018-1815-x	Excluded	Wrong patient population
23	Jaffar Kazmi (Schuerch)	2022	Handbook of Practical Immunohistochemistry: Frequently Asked Questions	10.1007/978-3-030-83328-2_16	Excluded	Wrong patient population
24	Sbiera (Fassnacht)	2019	Neuro Oncol	10.1093/neuonc/noz109	Included	-
25	Zhuang (Yu)	2024	BMC Med	10.1186/s12916-024-03573-y	Excluded	Wrong patient population
26	Casar-Borota (Lopes)	2024	Brain Pathol	10.1111/bpa.13302	Excluded	Wrong study design
27	Lamback (Gadelha)	2024	JCEM Case Rep	10.1210/jcemcr/luae143	Included	-
28	Nejo (Mukasa)	2019	J Neurosurg	10.3171/2018.6.JNS18729	Excluded	Wrong patient population
29	Watson (Bérubé)	2013	J Clin Invest	10.1172/JCI65634	Not assessed for eligibility	Wrong patient population
30	Gibbons (Higgs)	1996	Medicine (Baltimore)	10.1097/00005792-199603000-00001	Not assessed for eligibility	Wrong study design
31	Kristensen (Wesseling)	2019	Annals of Oncology	10.1093/annonc/mdz164	Not assessed for eligibility	Wrong study design
32	Prieto (Corbacho)	2023	Neuropathology	10.1111/neup.12858	Not assessed for eligibility	Wrong patient population
33	Ayers (Faradz)	2017	Human genomics	10.1186/s40246-017-0098-2	Not assessed for eligibility	Wrong patient population
34	Zhong (Liu)	2023	Cell Biology International	10.1002/cbin.11922	Not assessed for eligibility	Wrong patient population
35	Xu (Shen)	2021	BMC Endocrine Disorders	10.1186/s12902-021-00836-0	Not assessed for eligibility	Wrong patient population
36	De Arnaldo Silva Vellutini (De Oliveira)	2018	Surgical Neurology International	10.4103/sni.sni-319-17	Not assessed for eligibility	Wrong patient population
37	Legendre (Gilbert-Dussardier)	2017	American Journal of Medical Genetics, Part C: Seminars in Medical Genetics	10.1002/ajmg.c.31591	Not assessed for eligibility	Wrong patient population
38	He (Cao)	2024	Journal of Gastrointestinal Oncology	10.21037/jgo-23-979	Not assessed for eligibility	Wrong patient population
39	Correa (Freitas)	2022	Arquivos de Neuro-Psiquiatria	10.1590/0004-282X-ANP-2021-0261	Not assessed for eligibility	Wrong patient population

40	Obata (Yasuda)	2023	BMC Endocrine Disorders	10.1186/s12902-023-01373-8	Not assessed for eligibility	Wrong patient population
41	Weaver (Szyf)	2006	Proceedings of the National Academy of Sciences of the United States of America	10.1073/pnas.0507526103	Not assessed for eligibility	Wrong patient population
42	Jin (Zhang)	2016	Gene	10.1016/j.gene.2016.09.026	Not assessed for eligibility	Wrong patient population
43	Anand (Boulton)	2022	Nature	10.1038/s41586-021-04261-0	Not assessed for eligibility	Wrong study design
44	Balasubramanian (Crowley)	2017	American Journal of Medical Genetics, Part C: Seminars in Medical Genetics	10.1002/ajmg.c.31585	Not assessed for eligibility	Wrong patient population
45	Huang (Lou)	2016	Translational Gastroenterology and Hepatology	10.21037/tgh.2016.09.03	Excluded	Wrong patient population
46	Marrero-Rodríguez (Mercado)	2023	Archives of Medical Research	10.1016/j.arcmed.2023.102915	Excluded	Wrong study design
47	Terry (Perry)	2024	Endocr Pathol	10.1007/s12022-024-09829-w	Included	-
48	Casar-Borota (Lopes)	2025	Brain Pathol	10.1111/bpa.13302	Excluded	Wrong study design

1. Included Studies

Below is a table of the primary research studies reviewed and analyzed in this systematic review:

Table S2: List of Primary Studies Included in Systematic Review

Author	Year	Journal	DOI	Data Extractor	Date of Data Extraction	Confirmation of Eligibility
Casar-Borota (Trouillas)	2017	The American Journal of Surgical Pathology	10.1097/PAS.0000000000000908	EW	Jul-24	Yes
Guo (Wang)	2018	Frontiers in Oncology	10.3389/fonc.2018.00510	EW	Jul-24	Yes
Chen (Dahiya)	2019	Journal of Neuropathy and Experimental Neurology	10.1093/jnen/nlz040	EW	Jul-24	Yes
Sbiera (Fassnacht)	2019	Neuro-oncology	10.1093/neuonc/noz109	EW	Jul-24	Yes
Heaphy (Rodriguez)	2020	Modern Pathology	10.1038/s41379-020-0523-2	EW	Jul-24	Yes
Casar-Borota (Burman)	2021	The Journal of Clinical Endocrinology & Metabolism	10.1210/clinem/dgaa749	EW	Jul-24	Yes
Lu (Chen)	2021	Frontiers in Endocrinology	10.3389/fendo.2021.752361	EW	Jul-24	Yes
Alzoubi (Buttarelli)	2022	Endocrine Pathology	10.1007/s12022-021-09702-0	EW	Jul-24	Yes
Lamback (Gadelha)	2024	Journal of Clinical Endocrinology and Metabolism Case Reports	10.1210/jcemcr/luae143	EW	Jan-25	Yes
Terry (Perry)	2024	Endocrine Pathology	10.1007/s12022-024-09829-w	EW	Mar-25	Yes

All information regarding the study characteristics, extracted data for analysis, and risk of bias assessments from each included study are comprehensively provided within Tables 2–5 of the manuscript. This supplementary file explicitly references those tables for transparency.

Specifically:

- **Table 2**: Characteristics of included publications examining the prevalence of ATRX loss in human pituitary neuroendocrine tumours
- **Table 3**: Demographic and clinical patient data examined in the included publications
- **Table 4**: Quality assessment using the Joanna Briggs Institute (JBI) Critical Appraisal Checklist for Prevalence Studies
- **Table 5**: The Joanna Briggs Institute (JBI) Critical Appraisal Checklist for Risk of Bias for Case Reports that were identified
- **Table 6**: Demographic and histopathological characteristics of tumours exhibiting ATRX.

2. Statistical Methods

Fisher's exact tests were conducted to assess differences in categorical outcomes between groups, as reported in Tables 7A-C. Calculations were performed in Microsoft Excel using the hypergeometric distribution function:

=HYPGEOM.DIST(A, G, C, I, FALSE)

In this formula, values correspond to cells in a 2x2 contingency table as follows:

	Outcome Present	Outcome Not Present	Row Totals
Group 1	A	B	C
Group 2	D	E	F
Column Totals	G	H	I

This test evaluates the probability of observing the given distribution (or a more extreme one) under the null hypothesis of independence between categorical variables. All Fisher's exact test results relevant to Tables 7A-C are provided therein.
